# The Rise and Fall of SARS-CoV-2 Variants and Ongoing Diversification of Omicron

**DOI:** 10.3390/v14092009

**Published:** 2022-09-10

**Authors:** Tanner Wiegand, Artem Nemudryi, Anna Nemudraia, Aidan McVey, Agusta Little, David N. Taylor, Seth T. Walk, Blake Wiedenheft

**Affiliations:** 1 Department of Microbiology and Cell Biology, Montana State University, Bozeman, MT 59715, USA; 2 Clinical Research Department, Bozeman Health, Bozeman, MT 59715, USA

**Keywords:** SARS-CoV-2, Omicron, BA.4, BA.5, COVID-19, viral surveillance

## Abstract

In late December of 2019, high-throughput sequencing technologies enabled rapid identification of SARS-CoV-2 as the etiological agent of COVID-19, and global sequencing efforts are now a critical tool for monitoring the ongoing spread and evolution of this virus. Here, we provide a short retrospective analysis of SARS-CoV-2 variants by analyzing a subset (*n* = 97,437) of all publicly available SARS-CoV-2 genomes (*n* = ~11.9 million) that were randomly selected but equally distributed over the course of the pandemic. We plot the appearance of new variants of concern (VOCs) over time and show that the mutation rates in Omicron (BA.1) and Omicron sub-lineages (BA.2–BA.5) are significantly elevated compared to previously identified SARS-CoV-2 variants. Mutations in Omicron are primarily restricted to the spike and nucleocapsid proteins, while 24 other viral proteins—including those involved in SARS-CoV-2 replication—are generally conserved. Collectively, this suggests that the genetic distinction of Omicron primarily arose from selective pressures on the spike, and that the fidelity of replication of this variant has not been altered.

## 1. Introduction

All viruses, including SARS-CoV-2, accumulate mutations as they replicate and spread. Most of these changes have little or no impact on the transmissibility, disease severity, or effectiveness of current vaccines and diagnostics. However, selective pressures that act on phenotypes produced by random mutations efficiently enrich rare variants with enhanced viral fitness (e.g., replication and transmissibility). Initially, SARS-CoV-2 variants were named according to their geographic origin (e.g., Wuhan), but naming based on geography can be culturally insensitive, stigmatizing, and is often inaccurate. Thus, this naming scheme was quickly replaced by a unique combination of letters and numbers known as the Pangolin nomenclature (e.g., B.1.1.7), but alphanumerics are cumbersome and sometimes confusing for scientists and the public alike. Part of this confusion arises from the use of aliases, which are used to limit the growing string of letters and numbers that accumulate with successive differentiation [1]. With increasing frequency, we now complement the Pangolin nomenclature with the use of a single Greek letter. Omicron (B.1.1.529 or its alias BA.1) is the 15th letter of the Greek alphabet but only the fifth variant designated as a variant of concern (VOC) by the World Health Organization (WHO).

## 2. Omicron, the Newest Variant of Concern

**Figure 1:**
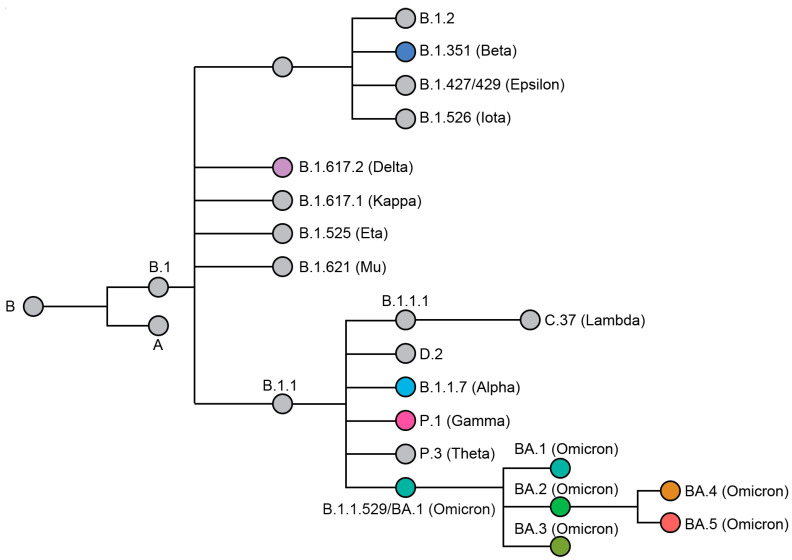
Phylogenetic relationship of named SARS-CoV-2 variants. Variants of concern (VOC) are represented by a colored node. The phylogenetic tree was adapted from data provided by NextStrain, CoVariants (i.e., covariants.org, http://covariants.org (accessed on 18 July 2022)), and Pangolin (i.e., cov-lineages.org, http://cov-lineages.org (accessed on 18 July 2022)) [1,8,13].

**Figure 2:**
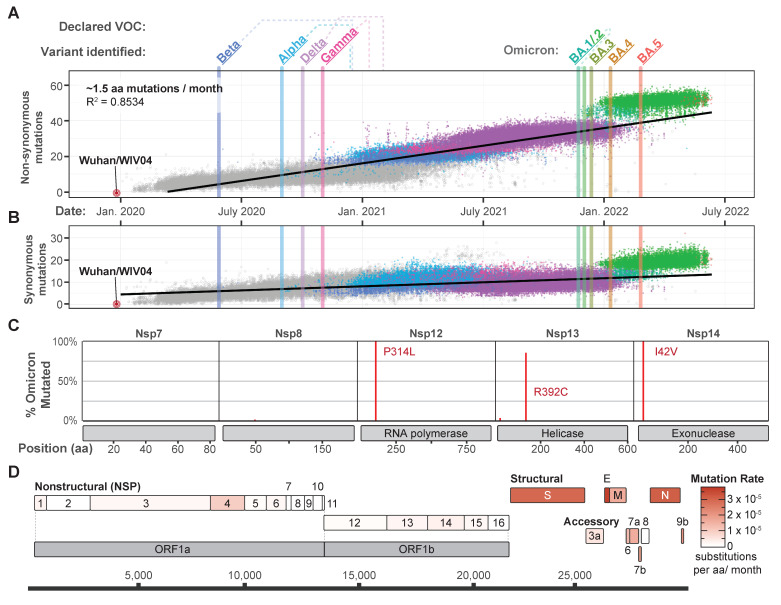
(**A**) Non-synonymous mutations acquired over time in 26 SARS-CoV-2 protein sequences extracted from 97,437 genomes from 19 December 2021, to 17 June 2022 (GISAID accessions available at: https://github.com/WiedenheftLab/Omicron (accessed on 18 July 2022) and DOI: https://doi.org/10.55876/gis8.220721mv (accessed on 18 July 2022)). Genomes included in this analysis are a random sampling of 11,336,176 million SARS-CoV-2 from GISAID, which were quality filtered using NextClade (“good” overall QC status) then sampled with the Filter utility in NextStrain (selecting up to 120 genome per country per month of the pandemic) [1,14]. Variants of concern (VOC) are shown in bold and are colored as in Figure 1. Vertical lines mark the date the first sequence for each lineage was identified. The time elapsed between first detection and VOC designation by the WHO is shown as dotted lines above the graph. Dots are colored similar to variant names, and grey circles represent non-VOC lineage genomes. A linear regression line is shown in black. Omicron variants deviate from the trend. (**B**) Synonymous mutations in the SARS-CoV-2 genomes shown in panel (**A**). (**C**) Non-synonymous mutations in the Omicron RNA replication proteins (*n* = 13,094 genomes) are shown as red lines on schematic representations of each protein, and frequencies of each mutation shown as vertical lines (red). Most non-synonymous mutations are found in less than 1% of Omicron sequences. (**D**) Schematic depiction of SARS-CoV-2 protein coding sequences, with each gene colored according to its respective amino acid mutation rate. These rates are normalized to account for the length of each protein (i.e., substitutions/amino acids in protein/month of the pandemic).

Omicron was first identified from a specimen collected on 9 November 2021 in South Africa and was designated as a VOC on 26 November [2]. This designation was based on the number of mutations (26–32) in the spike protein relative to previously sequenced isolates, as well as concerning epidemiological reports from South Africa [3,4]. Omicron has since diversified into five phylogenetically distinct sub-lineages (BA.1 to BA.5) (Figure 1) [1,5], which are frequently associated with vaccine breakthrough or reinfection of previously infected individuals [6,7]. Here, we take a step back and provide a short retrospective overview of how SARS-CoV-2 genomes have changed over the course of the pandemic. We analyze synonymous mutations that have no impact on the protein sequence and non-synonymous mutations that change the SARS-CoV-2 proteome (Figure 2A,B). Non-synonymous mutations, particularly those in the spike, tend to attract the most attention due to the prominent role of this protein in viral attachment and entry into the host. However, synonymous mutations also accumulate over time. Many of these mutations are neutral, but some alter expression patterns [8,9] or codon usage in ways that may improve fitness [10,11]. Collectively, we analyze a high-quality subset (*n* = 97,437) of all SARS-CoV-2 genomes (*n* = ~11.3 million) that were randomly selected but equally distributed over the course of the pandemic—starting with the Wuhan reference genome (2019) and ending with high-quality Omicron genomes from the GISAID database (17 June 2022) [12]. Overall, this analysis reveals a trend of increasing mutations over time, with a statistically significant jump in both synonymous and non-synonymous mutations in Omicron sub-lineages, relative to all the previous strains of the virus (*p* < 2 × 10^16^ and *p* < 2 × 10^16^, respectively; one-way ANOVA) (Figure 2A,B).

Based on the anomalous mutational profiles of Omicron viruses, we hypothesized that Omicron genomes have mutations that affect the fidelity of viral RNA replication machinery. To test this hypothesis, we analyzed the sequences of replication-associated proteins (i.e., Nsp7, Nsp8, Nsp12, Nsp13 and Nsp14) to determine whether they had acquired mutations that would be likely to affect the fidelity of replication (Figure 2C). This analysis revealed only one widespread mutation in the RNA-dependent RNA polymerase (i.e., P314L in Nsp12), which arose prior to the emergence of the Omicron and co-occurred with a spike mutation (D614G) that swept to global fixation in mid-2020 and is present in 97.8% of all the SARS-CoV-2 sequences we analyzed [15,16]. The only other widespread mutations we detected in the RNA replication components of Omicron were mutations in the RNA helicase (R392C in Nsp13) and a conservative amino acid substitution (i.e., I42V) in the 3′–5′ exoribonuclease protein (Nsp14) that is responsible for proof-reading (Figure 2C) [17,18]. Since these mutated residues are distal from the Nsp13 and Nsp14 active sites [19,20] and the P314L mutation in Nsp12 is not unique to Omicron variants, it seems unlikely that they are responsible for the increase in mutations acquired by Omicron lineage viruses. 

While the origins and conditions leading to the emergence of Omicron remain uncertain, some scientists suspect that Omicron arose in chronically infected, immunocompromised patients in which the immune response was too weak to clear the virus but strong enough to select for variants with increased fitness [21]. To identify other proteins that might be evolving under similar selective pressures, we evaluated mutational trends for each SARS-CoV-2 protein over the course of the pandemic (Figure 2D). Consistent with what has been observed previously, this analysis highlights the unique evolutionary signatures of the structural (i.e., S, E, M, N) and accessory proteins (i.e., 7a, 7b, 9, and 9b), which are evolving more rapidly than the non-structural proteins (NSP1-16) (Figure 2D) [5,22,23,24]. Such a concentration of mutations in some but not all loci is consistent with at least two different evolutionary scenarios. First, Omicron may have emerged from a dichotomy of strong purifying selection on NSP1-16 genes and strong diversifying selection on S, E, M, N, and accessory genes. Second, Omicron may represent a recombinant between a more ancestral (e.g., B1.1) lineage and a yet-to-be-discovered hyper-mutated virus (i.e., a virus with many mutations distributed more evenly across all loci) [25,26,27]. While there are examples of both scenarios among other viral emerging infectious diseases [28], the second scenario (recombination) is simpler and quicker compared to the necessary coincident rounds of opposing mutation-selection pressures of the first scenario. Additional sequencing of rare variants should help clarify Omicron’s evolutionary history.

## 3. The Future of SARS-CoV-2

Since most diagnostics target the N gene (PCR) or N protein (antigen), continued evolution of N is expected to complicate ongoing efforts to provide rapid and reliable diagnostics [29,30,31]. Similarly, changes in the spike will continue to have major impacts on the efficacy of natural or vaccine-induced immunity [6,7,32,33]. Increased incidence of breakthrough infections may indicate that a globally distributed and coordinated rollout of new vaccine cocktails that simultaneously targets the spike of several distinct variants [34,35,36,37] or multiple different viral genes that go beyond the spike [38,39] are necessary to establish durable immunity.

**Figure 3:**
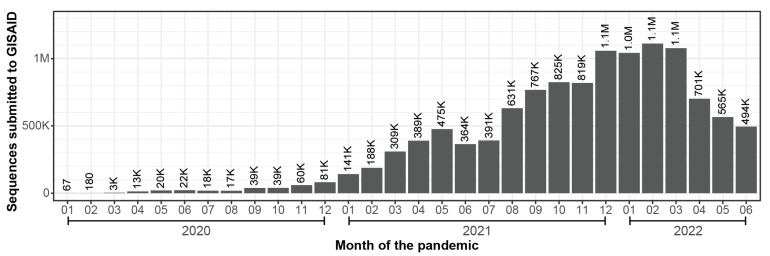
SARS-CoV-2 genomic sequences submitted to the GISAID data repository between January 2020 and June 2022 [12].

Accurately predicting the future is improbable, but it is safe to assume that the virus will continue to evolve and that we will continue to work our way through the Greek alphabet. However, at a time when Omicron variants are driving infection rates up (i.e., 3.3 million weekly cases on 5 June versus 6.7 million on 17 July) [40,41], viral surveillance is in rapid decline. Over a million SARS-CoV-2 genome sequences were deposited in GISAID in the months of November (2021) through February (2022), but submissions have dropped steadily over the last four months, and in June, the number of genomes contributed dipped below 500,000 (Figure 3) [12]. Wastewater surveillance helps fill in some of the gaps by providing community level assessments of the viral load [42], which is especially important as diagnostic testing moves out the clinic, where numbers are regularly tracked, to at-home testing, which is less sensitive and rarely reported to public health departments. There is no easy solution to this situation, but as infections increase, and tracking decreases, we are wandering blind into an uncertain future and we are at greater risk of being caught off guard by an unlucky role of the evolutionary dice, which may result in a new variant with distinct clinical outcomes.

## Data Availability

Accessions of GISAID sequences used in this work are available at https://github.com/WiedenheftLab/Omicron (accessed on 18 July 2022) and DOI: https://doi.org/10.55876/gis8.220721mv (accessed on 18 July 2022).
